# Screening of Germplasm Resources with Low-Phosphorus Tolerance During the Seedling Stage of Rice

**DOI:** 10.3390/plants14101543

**Published:** 2025-05-20

**Authors:** Mengru Zhang, Ye Wang, Zexin Qi, Qiang Zhang, Huan Wang, Chenglong Guan, Wenzheng Sun, Fenglou Ling, Zhian Zhang, Chen Xu

**Affiliations:** 1 Agronomy College, Jilin Agricultural University, Changchun 130118, China; 13848630735@163.com (M.Z.); 20220662@mails.jlau.edu.cn (C.G.);; 2 Institute of Agricultural Resources and Environment, Jilin Academy of Agricultural Sciences, Changchun 130033, China

**Keywords:** rice, screening of low-phosphorus tolerant germplasm, principal component analysis, cluster analysis

## Abstract

Rice is a globally important food crop, and phosphorus is an essential nutrient element for rice growth. In many of China’s arable lands, there is a deficiency in available phosphorus content. Therefore, screening and breeding rice germplasm resources that are tolerant to low phosphorus can enhance the growth capability of rice in low-phosphorus soils. This study set up treatments with two phosphorus concentrations: H_2_PO_4_^−^ at 0.18 mmol/L, referred to as normal phosphorus (NP), and H_2_PO_4_^−^ at 0.009 mmol/L, referred to as low phosphorus (LP). Using hydroponic methods, 156 different genotype rice germplasms were treated for 35 days, after which the morpho-physiological traits of the rice seedling shoots, root morphology, and material content were measured. An analysis of the coefficient of variation (CV) for low phosphorus tolerance coefficients across different rice germplasm resources revealed that 16 indicators had CVs greater than 10%, which can be used as criteria for screening rice varieties with low phosphorus tolerance at the seedling stage. The relevant indicators and low-phosphorus resistance characteristics of different rice varieties were comprehensively evaluated using principal component analysis, correlation analysis, membership function, and cluster analysis methods. The results indicate that the principal component analysis transformed 23 indicators into 5 comprehensive indicators, with a cumulative contribution rate of 86.947%. The *D* value was evaluated in a comprehensive evaluation of low-phosphorus resistance, and 156 rice germplasm resources were divided into four types by cluster analysis. A scatter plot was created using the comprehensive phosphorus efficiency values of different rice germplasms under normal phosphorus and low phosphorus conditions. Through further verification, the germplasms with strong low-phosphorus tolerance finally selected through comprehensive screening were Y3-14, Y3-35, Y3-21, Jinnongda 705, Changjing 625, and Jinnongda 873. The germplasms with poor low-phosphorus tolerance were Jijing 338, Jingu 981, Tong 35, Y3-31, and Longdao 20.

## 1. Introduction

Rice is one of China’s important food crops and the most crucial staple food crop in Asia [1]. Ensuring and increasing rice production are crucial for stabilizing global food security [2]. Phosphorus (P) is an essential nutrient element for crops [3]. It is a major component of ATP, a molecule that provides energy to plants and is used in processes such as photosynthesis, protein synthesis, and respiration. Studies have shown that phosphorus can promote root growth and influence earliness, stalk strength, crop quality, and disease resistance [4]. The global demand for phosphate fertilizers continues to rise, while the global commercial phosphate reserves are estimated to become depleted within the next few decades [5]. Soil phosphorus-deficiency stress is one of the major limiting factors for the growth and yield of many crops worldwide, including rice [6]. Crops alone consumed 1.07 million tons of P_2_O_5_, with a consumption of 24.3 kg of P_2_O_5_ per hectare [7]. The limitation of yield by phosphorus availability can be overcome by applying phosphate fertilizers or cultivating rice varieties that thrive in low-phosphorus soil conditions. However, the extensive application of phosphate fertilizers not only increases production costs and import demands but also leads to water pollution due to fertilizer runoff [8]. Phosphorus is essential for rice growth, but the majority of phosphorus fertilizers applied to the soil form insoluble phosphorus compounds by binding with ions such as Ca^2+^, Fe^2+^, Fe^3+^, and Al^3+^. As a result, the utilization rate of phosphorus fertilizers during the rice growing season does not exceed 30% [9], leading to the accumulation of a significant amount of phosphorus in the soil in an ineffective form [10]. Causing the waste of phosphorus resources, soil compaction, and water pollution, fertilization is not the optimal solution to low-phosphorus stress [8]. There is currently no known substitute for phosphate fertilizers that can support plant growth and development [11]. Therefore, screening and cultivating low-phosphorus-tolerant varieties is of significant importance, serving as one of the key measures for alleviating the depletion of phosphate rock, reducing the application of phosphate fertilizers, addressing the deficiency of available phosphorus in soil, maintaining relatively stable yields, and protecting the agricultural ecological environment.

In recent years, research on plants tolerant to low-phosphorus stress has received increasing attention. Screening and cultivating crop varieties with low-phosphorus tolerance have become a common concern for researchers in plant nutrition and crop genetic cultivation. The screening method directly influences the success or failure of a screening experiment. According to the different cultivation media, the screening methods can be divided into the soil culture method, sand culture method, and water culture method [12]. Each method has its own advantages and disadvantages. Soil culture mainly involves pot planting and field screening. These methods make it difficult to promptly obtain the growth status of plant roots, thus precluding direct observation and measurement of the root system. Additionally, the composition of soil is complex, containing numerous uncontrollable factors [13]. The entire sand culture system is not only difficult to obtain but also complex and time-consuming to operate, which is why it has not been widely adopted for screening crop varieties with low-phosphorus tolerance [14]. Hydroponics allows for the precise control of the element content in the nutrient solution, is simple and quick, and ensures consistent experimental conditions, but it is not suitable for screening crops throughout their entire growth periods [15]. Regarding the screening of low-phosphorus-tolerant genotypes in crops, previous researchers have already carried out extensive work. Liu Ya et al. discovered that low-phosphorus stress can induce an increase in acid phosphatase activity in rice roots [16]. The organic acids secreted by the roots undergo anion exchange or competitive adsorption with insoluble phosphates, thereby releasing or dissolving the insoluble phosphates. Under low-phosphorus stress conditions, the growth status of the plants can directly reflect their tolerance to phosphorus. Further studies have shown that, under low-phosphorus stress during the seedling and jointing stages, the relative phosphorus uptake of low-phosphorus-tolerant inbred lines is significantly higher than that of sensitive inbred lines. The low-phosphorus tolerance mechanism is primarily characterized by high absorption efficiency, with utilization efficiency contributing little to phosphorus efficiency [17]. Under low-phosphorus conditions, some plants can grow normally, while others experience inhibited growth or even death. Different plants exhibit varying efficiencies in phosphorus utilization, primarily manifested in phosphorus allocation, utilization, transport, and absorption [18]. Plants have evolved various mechanisms to adapt to low-phosphorus conditions, such as upregulating root development and promoting lateral root growth [19,20]; secreting more organic acids from their roots [21]; promoting mycorrhizal formation [22]; producing acid phosphatases to combat low-phosphorus stress [23,24]; and altering their metabolic pathways [25,26]. Liu Yuan et al. explored the changes in plant height, dry weight, and phosphorus content of soybeans under different phosphorus levels based on the low-phosphorus tolerance coefficient of acid phosphatase [27]. They found significant differences in low-phosphorus tolerance among different soybean genotypes and successfully screened out soybean germplasm with low-phosphorus tolerance. The production of rice also faces the issue of phosphorus deficiency. There is scarce reporting on research regarding the evaluation of phosphorus efficiency in different rice germplasm resources. This study selected 156 rice germplasm resources and comprehensively evaluated multiple indicators during the seedling stage, including the plant height, root length, aboveground fresh weight, underground fresh weight, leaf area, aboveground phosphorus content, underground phosphorus content, aboveground dry weight, and underground dry weight. The research delved into the biological traits and phosphorus utilization efficiency of rice germplasm under low-phosphorus conditions, aiming to identify indicators of low-phosphorus tolerance and rice germplasm resources with low-phosphorus tolerance during the seedling stage. This is not only of great significance for improving the efficiency of phosphate fertilizer utilization and promoting sustainable agricultural development but also provides a theoretical basis for utilizing these excellent germplasm resources in genetic cultivating research.

## 2. Results

### 2.1. Effects of Low-Phosphorus Stress on Single Index of Rice Seedlings with Different Genotypes

Table S1 shows the performance of 156 rice germplasms under two phosphorus concentrations. The variation coefficients of different rice germplasm resources under NP were 13.06–39.32%, and the variation coefficient under LP was 14.15–38.66%, indicating that the indices of 156 different rice germplasms have varying degrees in the NP and LP environments. The differences between different indicators in the NP and LP environments were analyzed, and the following results were obtained: Compared with those under NP, the root length, root–crown ratio, and root surface area of most rice seedlings significantly increased (*p* ≤ 0.001), and the root volume under LP was also significantly higher than that under normal-phosphorus conditions (*p* ≤ 0.05), indicating that the root development of plants is promoted under LP. Plants under LP cope with the lack of phosphorus by adjusting the morphological structure of their roots, such as through the growth of the root system and the expansion of the root surface area. Other indicators decreased under low-phosphorus conditions, so screening under LP is feasible. Under normal phosphorus levels, the plant height; SPAD; fresh weight above ground; dry weight underground; total dry weight per plant; aboveground phosphorus content; underground phosphorus content; single-plant phosphorus content; aboveground, underground, and single-plant phosphorus accumulation; aboveground phosphorus utilization efficiency; single-plant phosphorus utilization efficiency; and average diameter were significantly increased (*p* ≤ 0.001). The values of these parameters under low phosphorus were 61.8%, 37.7%, 37.7%, 15.2%, 15.2%, 85.7%, 103.1%, 92.3%, 51.2%, 87.8%, 130.9%, 35.4%, 87.5%, and 9.9% higher than under the low phosphorus level, respectively. The underground fresh weight, total fresh weight of a single plant, underground phosphorus utilization efficiency, and acid phosphatase also increased significantly (*p* ≤ 0.05); they were 18.5%, 14.2%, 87.5%, and 9.9% higher than under the low phosphorus level, respectively. The leaf area and aboveground dry weight also increased, being 10.1% and 8.6% higher than under the low phosphorus level, respectively, with no significant difference, indicating that the growth of various traits in the aboveground parts of rice was inhibited under low-phosphorus conditions.

### 2.2. Analysis of Low-Phosphorus Tolerance Coefficients for Various Indices in Different Rice Germplasms

There were different degrees of changes in the indices for 156 different rice germplasms in NP and LP environments. The low-phosphorus resistance coefficient (relative value) that can reflect the low-phosphorus resistance ability of the varieties is calculated as the basis for judging the low-phosphorus resistance characteristics of rice. The low-phosphorus coefficients for indicators such as the plant height, aboveground fresh weight, SPAD, aboveground and underground dry weight, total dry weight per plant, phosphorus content in aboveground and underground parts, phosphorus accumulation per plant, phosphorus utilization efficiency per plant, and average diameter were all less than 1. The total fresh weights per plant of 38 rice germplasms had a low-phosphorus tolerance coefficient greater than 1. The root–shoot ratios of 45 rice germplasms had a low-phosphorus tolerance coefficient greater than 1. The acid phosphatase of 58 rice germplasms had a low-phosphorus tolerance coefficient greater than 1. Comprehensively, the low-phosphorus conditions inhibit the growth of various traits in the upper ground, and the phosphorus content of rice under low phosphorus is lower than that in normal environments. The root lengths, root surface areas, and root volumes of most of the rice germplasms had a low-phosphorus tolerance coefficient greater than 1; low phosphorus can stimulate the root development of plants. In order to adapt to low-phosphorus conditions, the plant roots become thin and long. As shown in Table 1, the coefficients of variation of the low-phosphorus resistance coefficient of each index rank as follows: relative phosphorus use efficiency per plant > relative aboveground fresh weight > relative total dry weight per plant > relative acid phosphatase > relative underground dry weight > relative aboveground dry weight > relative root length > relative phosphorus accumulation per plant > relative underground phosphorus content > relative total fresh weight per plant > relative aboveground phosphorus use efficiency > relative underground phosphorus accumulation > relative aboveground phosphorus accumulation > relative plant height > relative aboveground phosphorus content > relative underground fresh weight > relative underground phosphorus use efficiency > relative root surface area > relative average diameter > relative leaf area > relative SPAD > relative root–shoot ratio > relative root volume. Among them, the coefficient of variation relative to the utilization efficiency of phosphorus in a single plant was the largest, at 39.97%. The coefficient of variation relative to root volume was the smallest, at 5.02%. The relative value variation coefficients of 16 indicators were above 10%. It can be seen that there were large differences in the low-phosphorus tolerance characteristics of different rice germplasms. There were extensive genetic variations in 156 germplasms at low phosphorus and normal phosphorus levels. Given that the above indicators show large variations in different phosphorus environments, these indicators are screening indicators that can identify rice with good low-phosphorus resistance.

### 2.3. Correlation Analysis of Low-Phosphorus Tolerance Index Among Different Genotypes of Rice Germplasm Indicators

**Figure 1:**
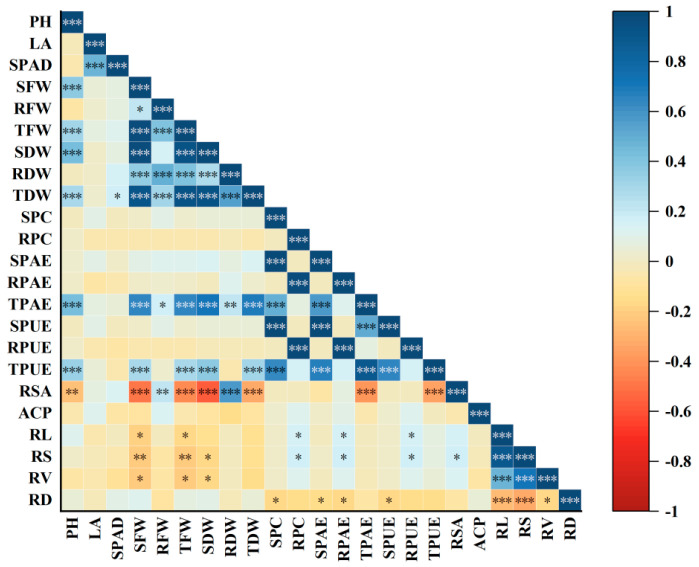
There was correlation between a normal P level and low P level in the seedling stage of 156 rice materials. Note: There is a column of values on the right (ranging from 1 to −1). This typically represents the numerical values or levels corresponding to different colors. The color scale from −1 to 0 indicates a negative correlation between indicators, where a smaller value signifies a more significant negative correlation; the color scale from 0 to 1 indicates a positive correlation between indicators, where a larger value signifies a more significant positive correlation. *** Significant correlation at 0.001 level (bilateral). ** Significant correlation at 0.01 level (bilateral). * Significant correlation at 0.05 level. Plant height: PH; leaf area: LA; SPAD: SPAD; aboveground fresh weight: SFW; belowground fresh weight: RFW; total fresh weight per plant: TFW; aboveground dry weight: SDW; belowground dry weight: RDW; total dry weight per plant: TDW; aboveground phosphorus content: SPC; belowground phosphorus content: RPC; aboveground phosphorus accumulation: SPAE; belowground phosphorus accumulation: RPAE; total phosphorus accumulation per plant: TPAE; aboveground phosphorus utilization efficiency: SPUE; belowground utilization efficiency: RPUE; total utilization efficiency per plant: TPUE; root–shoot ratio: RSA; acid phosphatase: ACP; root length: RL; root surface area: RS; root volume: RV; average diameter: RD.

In order to further screen out the identification indices for evaluating the low-phosphorus tolerance of rice, the correlation analysis of the measured indices was carried out. As shown in Figure 1, the low-phosphorus tolerance coefficient of plant height (PH) was related to the aboveground fresh weight (SFW) (r = 0.908 ***), total fresh weight per plant (TFW) (r = 0.912 **), and aboveground dry weight (SDW) (r = 0.925 **), and leaf area (LA) was positively correlated with SPAD (r = 0.923 ***). The root–shoot ratio (RSA) was related to the aboveground fresh weight (SFW) (r = −0.847 **), total fresh weight per plant (TFW) (r = −0.872 **), aboveground dry weight (SDW) (r = −0.903 **), and total dry weight per plant (TDW). The root length (RL) was positively correlated with the root surface area (RS) (r = 0.894 **) and root volume (RV) (r = 0.907 ***). Because there is a certain degree of correlation among the traits, the information provided by them overlaps, and the role of each trait in identifying low-phosphorus tolerance in rice is not the same. Therefore, we cannot directly use one or several indices to evaluate the low-phosphorus tolerance of germplasms, but we need to evaluate the low-phosphorus tolerance of different germplasms through a comprehensive analysis.

### 2.4. Principal Component Analysis of Different Genotypes of Rice Germplasm

A principal component analysis was conducted based on the low-phosphorus tolerance coefficients of 16 individual indicators with a coefficient of variation (CV) greater than 10%. From Table 2 and Table 3, it can be seen that the weight coefficients of the 16 major low-phosphorus resistance characteristic indices on each principal component, namely the eigenvector, among which the contribution rate of the first principal component is the most significant, and its key characteristic vectors are the plant height and phosphorus accumulation amount per plant. The weight coefficients are the highest, 0.141 and 0.139, respectively. This shows that plant height and phosphorus accumulation amount per plant are important indicators and good for studying low-phosphorus resistance characteristics in rice; the characteristic value was 6.78, and the contribution rate reached 42.377%. The second principal components, the loads of the underground dry weight and root crown ratio, were higher, at 0.338 and 0.311, respectively. The characteristic value was 2.781, accounting for 17.384% of the original indicator’s information. These mainly reflect root system factors, indicating that rice will cope with a low-phosphorus environment by adjusting its root system’s morphology and structure, making the root system thin and long and increasing the underground dry weight. In addition, the root–crown ratio is increased. The root–crown ratio is increased because low-phosphorus stress prompts the plants to distribute more assimilation products to the root. The third principal component is the total fresh weight of a single plant as the main load, and the weight coefficient was 0.418, reflecting 11.521% of the total information volume. The fourth principal component uses the aboveground phosphorus utilization efficiency as the main load, the weight coefficient was 0.586, and the eigenvalue was 1.313, reflecting 8.207% of the total information volume. The fifth main component was the main load with underground fresh weight. The cumulative contribution rate of the first five principal components reached 86.947%. According to the size of the contribution rate, we know the relative importance of the comprehensive indicators. These five independent comprehensive indicators were used to analyze the phosphorus efficiency characteristics of the different rice.

### 2.5. Comprehensive Analysis of Low Phosphorus Tolerance of Different Genotypes of Rice Germplasm

Based on the results of the principal component analysis, Formula (2) was used to calculate the membership function value μ(X) of each comprehensive index. As shown in Table S2, under low-phosphorus stress conditions, in comprehensive index Z1, the μ(X1) value of V147 was the smallest, at 0, which means that the low-phosphorus tolerance of V147 was the weakest in the Z1 index. The V76 variety had the largest μ(X1) value, at 1, and it had strong low-phosphorus resistance in Z1. Similarly, for the comprehensive indicators Z2, Z3, Z4, and Z5, the varieties with the smallest μ(X) values of V93, V56, V136, and V38 and the varieties with the largest μ(X) value of V80, V118, V46, and V88 were found, respectively. This shows that there are differences in the identification results for rice low-phosphorus resistance when using different comprehensive indicators. Using Formula (3) and combined with the contribution rate of each comprehensive indicator, it was calculated that the weight W of the five comprehensive indicators was 48.7%, 19.9%, 13.3%, 9.4%, and 8.7%. These weights reflect the importance of different comprehensive indicators in evaluating the specific gravity of low-phosphorus tolerance in rice. In order to more accurately evaluate the low-phosphorus resistance of different rice varieties, the comprehensive evaluation value *D* of the five comprehensive indicators was further calculated using Formula (4). Based on the size of the *D* value, it can be clearly seen that, the larger the *D* value, the stronger the ability to tolerate low phosphorus. Among the varieties, V78, V80, V131, V73, etc., had a strong ability to tolerate low phosphorus. On the contrary, V59, V58, V56, V6, and others had a weak ability to tolerate low phosphorus. Through the calculation and analysis of the comprehensive evaluation value *D*, it will be possible to more intuitively understand the differences in the tolerance of low-phosphorus characteristics among different rice varieties.

### 2.6. Cluster Analysis of Different Genotypes of Rice Germplasm

**Figure 2:**
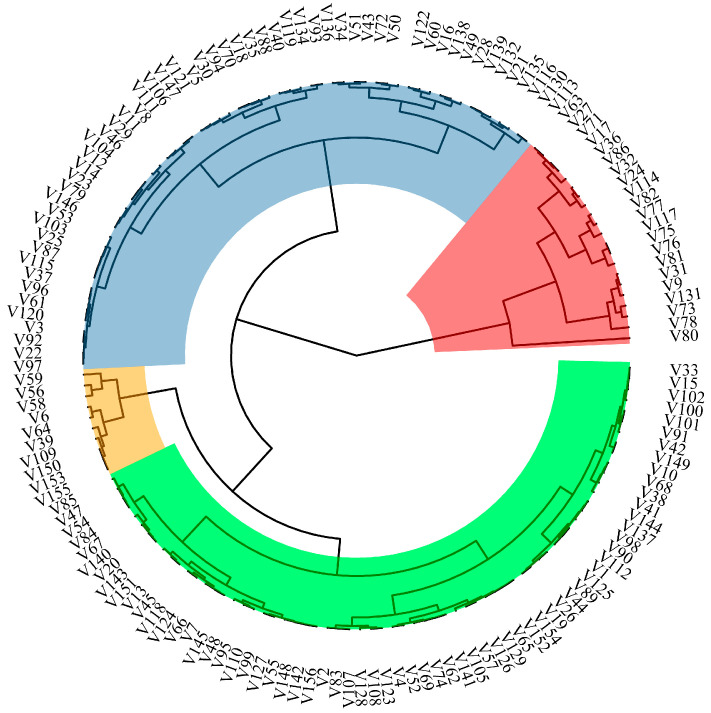
*D*-value cluster map of 156 rice varieties with different genotypes at the seedling stage. Note: Red: Low-phosphorus-tolerant strong germplasm. Blue: Low-phosphorus-tolerant relatively strong germplasm. Green: Low-phosphorus-tolerant relatively weak germplasm. Yellow: Low-phosphorus-tolerant weak germplasm.

The sum of squared distances method was used to cluster the comprehensive evaluation value *D* calculated from five comprehensive indicators of low-phosphorus tolerance. As shown in Figure 2, the results divided 156 rice germplasms of the experiment into 4 categories. The first category includes 20 rice germplasms such as Jinongda 705, Changjing 625, Jinongda 873, and Longdao 1001. The *D* value ranged from 0.7145 to 0.5677, and they had strong low-phosphorus tolerance. The second category includes 59 rice germplasms such as Q5-17, Dongdao 607, Q3-12, and Q5-8. The *D* value ranged from 0.5566 to 0.3999, and they had strong tolerance to low phosphorus. The third category includes 67 rice germplasms such as Y3-42, Songliang 23, Muyudao 100, and Q3-9. The *D* value ranged from 0.3833 to 0.3475, indicating a relatively weak ability to tolerate low phosphorus. The fourth category includes 10 rice germplasms such as Y3-31, Tong 35, Jingu 981, and Longdao 20. The *D* value ranged from 0.2348 to 0.1505, indicating weak low-phosphorus tolerance.

### 2.7. Comprehensive Value Analysis of Phosphorus Efficiency of Rice Germplasm at Seedling Stage with Different Phosphorus Concentrations

**Figure 3:**
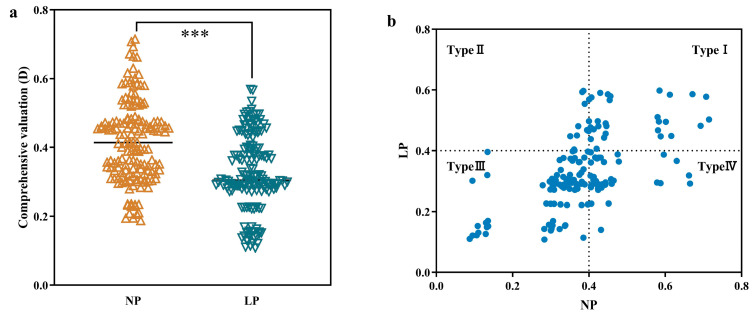
Scatter plot of phosphorus efficiency of different rice germplasm resources under different phosphorus levels. Note: (**a**) Phosphorus comprehensive efficiency values of 156 different rice germplasms under two phosphorus levels. (**b**) Scatter plot of the comprehensive phosphorus efficiency values (*D* values) of different rice resources under NP and LP levels. *** Significant at 0.001 level (bilateral).

To further verify the above results, through a comprehensive analysis of the membership function, the comprehensive phosphorus efficiency values of different rice germplasms under NP and LP levels were obtained to evaluate the advantages and disadvantages of different germplasm resources in terms of phosphorus efficiency (Table 4). The comprehensive phosphorus efficiency values under NP and LP levels were analyzed. From Figure 3a, it can be seen that there were significant differences in the comprehensive phosphorus efficiency values of 156 rice germplasms under NP and LP levels (*p* ≤ 0.001). Scatter plots were drawn using the comprehensive phosphorus efficiency values (*D* values) of different rice resources under NP and LP levels, and the different rice resources were divided into four categories (Figure 3b). Type I is the type with strong tolerance to low phosphorus under normal-phosphorus conditions and strong tolerance to low phosphorus under low-phosphorus conditions (NP and LP > 0.4). Type II is the type with weak tolerance to low phosphorus under normal-phosphorus conditions and strong tolerance to low phosphorus under low-phosphorus conditions (NP < 0.4 and LP > 0.4). Type III is the type with weak tolerance to low phosphorus under normal-phosphorus conditions and weak tolerance to low phosphorus under low-phosphorus conditions (NP and LP < 0.4). Type IV is the type with strong tolerance to low phosphorus under normal-phosphorus conditions and weak tolerance to low phosphorus under low-phosphorus conditions (NP > 0.4 and LP < 0.4). Among them, there are 35 rice germplasms in Type I, 5 rice germplasms in Type II, 71 rice germplasms in Type III, and 45 rice germplasms in Type IV.

**Figure 4:**
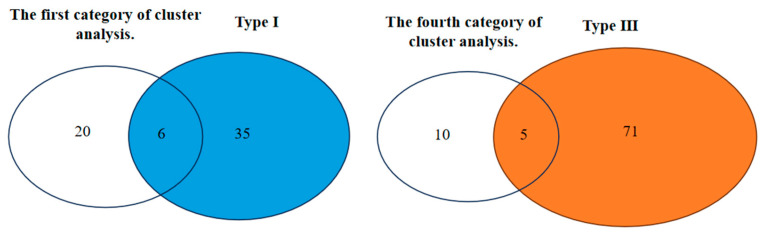
Intersection diagram of the results of two classification methods.

It is concluded from clustering that there are 20 germplasms with strong tolerance to low phosphorus in the first category. As shown in Figure 4, there are 35 germplasms with NP and LP > 0.4 under two different phosphorus levels. Both sets of results include a total of six germplasms: V17, V71, V75, V78, V80, and V131. A comprehensive analysis shows that the germplasms with strong tolerance to low phosphorus are Y3-14, Y3-35, Y3-21, Jinnongda 705, Changjing 625, and Jinnongda 873. There are 10 germplasms with poor low-phosphorus tolerance derived from clustering. There are 71 germplasms with NP and LP < 0.4 under two different phosphorus levels. Both sets of results include a total of five germplasms: V39, V56, V58, V59, and V64. Based on the comprehensive analysis, the germplasms with poor low-phosphorus tolerance were determined to be Jijing 338, Jingu 981, Tong 35, Y3 -31, and Longdao 20.

## 3. Discussion

Exploring the potential of crop phosphorus utilization and screening for low-phosphorus-tolerant varieties are effective approaches to enhance crop phosphorus use efficiency and reduce environmental pollution. The most direct and objective method of screening for low-phosphorus-tolerant genotypes is to cultivate them in phosphorus-deficient soil and evaluate their economic yield. However, the variability in various physical and chemical properties of the soil increases the difficulty of controlling the experimental conditions. Research indicates that, compared to field trials, screening for low-phosphorus tolerance resources during the rice seedling stage can shorten the screening time and reduce the workload. Therefore, hydroponics is a feasible method for screening low-phosphorus-tolerant crops in the short term [28]. Both the relative values of individual indicators within the same germplasm and the relative values of the same indicator across different germplasms demonstrate notable disparities. To address the inadequacy of single indicators in evaluating rice’s tolerance to low phosphorus, multivariate statistical methods were further employed for analysis and assessment, utilizing fewer comprehensive indicators to replace the original multitude of indicators. The comprehensive scores were calculated using a principal component analysis and the membership function method, and the rice germplasms were classified through a cluster analysis, thereby significantly improving the efficiency of screening for low-phosphorus-tolerant germplasms. The use of comprehensive indicators to evaluate the stress resistance of crops has achieved certain results in alfalfa [29], peanuts [30], and rice [31]. The existing research results indicate that the rational selection of trait indicators is crucial for the identification of low-phosphorus tolerance in crops. The internal utilization efficiency of phosphorus in low-phosphorus solutions during the seedling stage can be utilized as a screening criterion for identifying low-phosphorus-tolerant rice varieties [32]. Studies have also shown that the increases in the root length, root weight, root-to-shoot ratio, fresh weight, and total dry weight under phosphorus deficiency stress at the seedling stage can serve as indicators of rice tolerance to low-phosphorus stress [33]. In early maize field trials, it was demonstrated that shoot weight is the plant parameter most sensitive to phosphorus deficiency [34]. The coefficient of variation among different indicator varieties can reflect the sensitivity of the varieties to low-phosphorus stress. The greater the coefficient of variation, the larger the disparity in low-phosphorus stress’ impact among varieties and the higher the contribution to their low-phosphorus tolerance [35]. This study shows that, under low-phosphorus conditions, the coefficient of variation (CV) for 23 indicators in rice, including the plant height, leaf area, fresh weight of aboveground and underground parts, dry weight of aboveground and underground parts, plant phosphorus content, plant phosphorus accumulation, root-to-shoot ratio, acid phosphatase, and root length, all exceed 10%. This indicates that low phosphorus increases the variability in morpho-physiological traits and phosphorus utilization and absorption among rice germplasms. These indicators can be used as screening criteria for identifying low-phosphorus tolerance in rice.

There are significant differences in phosphorus absorption and utilization among different genotypes of the same crop, cultivating crop varieties tolerant to low phosphorus [36]. This study found that the plant height, leaf area, and aboveground dry weight of low-phosphorus-tolerant germplasms such as Jinongda 705, Changjing 625, and Jinongda 873 were higher than the mean values of these indicators, while those of low-phosphorus-sensitive germplasms such as Jijing 338, Jingu 981, and Tong 35 were lower than the mean values of these indicators This is because under low-phosphorus conditions, low-phosphorus-tolerant germplasms are capable of producing more biomass with lower internal phosphorus content. This adaptive advantage ensures that under low-phosphorus stress, the physiological metabolism of low-phosphorus-tolerant rice genotypes is less inhibited compared to low-phosphorus-sensitive genotypes. Ultimately, the reduction in aboveground biomass was significantly smaller compared to low-phosphorus-sensitive rice. This study, as indicated in Section 2.1, found that compared to low phosphorus conditions, the plant height, SPAD, dry weight, phosphorus content, and phosphorus accumulation of 156 rice germplasm resources significantly increased (*p* ≤ 0.001) by 39.2%, 37.3%, 33.3%, 32.2%, and 42.3%, respectively, under normal phosphorus conditions. This study ultimately revealed that under low-phosphorus conditions, the aboveground plant height, SPAD, dry weight, phosphorus content, and phosphorus accumulation of rice seedlings were all lower than the corresponding indicators under normal-phosphorus conditions. Studies have shown that low phosphorus promotes root length increase in certain rice varieties [37]. This study, as outlined in Section 2.1, indicates that under normal phosphorus conditions, the average root length, root surface area, and root diameter of 156 rice germplasm resources are 14.335 cm, 2.677 cm^2^, and 0.535 mm, respectively. Under low phosphorus conditions, the average root length and root surface area are 16.058 cm, 2.934 cm^2^, and 0.487 mm, respectively. An analysis of variance revealed that under low phosphorus conditions, the root length and root surface area of rice significantly increased (*p* ≤ 0.001), while the root diameter significantly decreased (*p* ≤ 0.001). This is primarily because, when plants face phosphorus-deficient conditions, the substances synthesized in the aboveground parts are transported more to the roots, resulting in a much greater inhibition of growth in the aboveground parts compared to the roots [38]. The elongation of root systems facilitates an increased contact area with phosphorus, thereby enhancing the efficiency of phosphorus absorption. The low-phosphorus tolerance performance of rice is a complex process. Phosphorus deficiency simultaneously stimulates root growth, leading to an increase in the fresh weight of the underground parts. It also promotes an increase in acid phosphatase activity in the rice roots. The organic acids secreted by the roots engage in anion exchange or competitive adsorption with insoluble phosphates, thereby releasing or dissolving these insoluble phosphates, which enhances the phosphorus utilization efficiency. In this experiment, by analyzing the correlation magnitudes among various measured indicators, a principal component analysis was employed to transform 24 indicators into 5 comprehensive indicators, with a cumulative contribution rate of 86.947%. The membership function method was used to calculate the comprehensive scores of each rice germplasm, and a cluster analysis was conducted based on the comprehensive evaluation value *D* for low-phosphorus tolerance, classifying the tested rice germplasms into four categories. Through further verification and final screening, the six germplasms with strong tolerance to low phosphorus were found to be Y3-14, Y3-35, Y3-21, Jinnongda 705, Changjing 625, and Jinnongda 873. The five germplasms with poor tolerance to low phosphorus were Jijing 338, Jingu 981, Tong 35, Y3-31, and Longdao 20. Currently, there is limited research on the genetic and molecular mechanisms related to the internal phosphorus utilization efficiency of low-phosphorus-tolerant rice genotypes [39,40].

## 4. Materials and Methods

### 4.1. Experimental Materials

Varieties suitable for cultivation in Jilin Province were selected and bred along with the 156 different rice germplasm resources currently under development (Table S2), with the germplasm resource type being japonica rice. These materials were provided by the Rice Research Institute of Jilin Agricultural University and used as experimental materials.

### 4.2. Experimental Design

The experiment was executed within the greenhouse facilities of Jilin Agricultural University from May to October 2023. Based on a comprehensive review of the relevant literature, preliminary experiments on phosphorus concentration were conducted, thereby determining 0.009 mmol/L of H_2_PO_4_^−^ as the low-phosphorus treatment level [41]. Prior to sowing, rice seeds underwent a disinfection procedure, first immersed in 30% hydrogen peroxide (H_2_O_2_) solution for 30 min, followed by 24 h of soaking in a 0.1% sodium chlorate (NaClO_3_) solution. Subsequently, the seeds were transferred to a thermostatic and humidity-controlled chamber to initiate the germination process at a constant temperature of 35 °C. This study employed a hydroponic cultivation system. In the initial stage of rice cultivation, the rice seedlings were cultivated in distilled water for five days, followed by continued cultivation in a diluted one-half standard concentration Kimura B nutrient solution for seven days. Thereafter, the rice seedlings were transferred to a diluted standard concentration of Kimura B nutrient solution, the detailed composition of which is shown in Table 5. After growing in the diluted standard nutrient solution for seven days, they were treated with diluted Kimura B nutrient solutions of different phosphorus concentrations. The experiment established two different phosphorus treatment levels: 0.18 mmol/L of H_2_PO_4_^−^ after dilution as the normal phosphorus treatment, denoted as (NP), and 0.009 mmol/L of H_2_PO_4_^−^ after dilution as the low phosphorus treatment, denoted as (LP). All other nutritional components remained consistent across the treatments. Germinated seeds were sown in seedling trays at a density of three plants per planting hole. The trays were positioned on plastic turnover boxes (dimensions: 560 mm × 420 mm × 190 mm, volume: 40 L) filled with the respective nutrient solutions. Each experimental treatment was replicated three times to ensure statistical reliability. Throughout the cultivation period following the phosphorus treatment, the nutrient solution was replaced every three days. In the low-phosphorus nutrient solution, as the amount of K_2_HPO_4_ was lower, the missing K^+^ was supplemented with KCl to prevent differences in the content of K^+^. To maintain optimal growth conditions, the pH of the nutrient solution was adjusted daily to a range of 5.5–6.0 using 0.1 mmol/L hydrochloric acid (HCl) or sodium hydroxide (NaOH) solutions. Additionally, to mitigate potential edge effects, the positions of the trays were systematically rearranged during each nutrient solution replacement. Plant samples were collected 35 days post-treatment for subsequent analyses.

### 4.3. Determination of Related Indicators

(1) After sampling the plant, the plant height and leaf area were measured. The total fresh weight of a single rice plant was measured by drying the water absorbed by its root system. The rice plants were then divided into aboveground and underground parts, and the fresh weights of the aboveground and underground portions were measured separately. Then, they were put into an oven and dried until the weight was constant; subsequently, the dry weight on the ground, the dry weight of the root, the total dry weight of the single plant, and the root–crown ratio were calculated. Three plants of each variety were randomly selected as biological replicates.
(2) The root system was carefully scanned into images using a digital scanner and stored on a computer to quantitatively analyze the total root length, root surface area, root system volume, and root diameter. Three plants were randomly selected for each variety as replicates.
(3) Determination of phosphorus content: After weighing and crushing the aboveground and underground parts of the plants, the plant samples were digested with concentrated H_2_SO_4_ and 30% H_2_O_2_, and the volume was fixed to 100 mL; the total phosphorus content in each part of the plant was determined by the molybdenum antimony colorimetric method [42].
(4) Root acid phosphatase: This was measured by determining the acid phosphatase activity through colorimetry at a wavelength of 400 nm [43].
(5) SPAD: Five leaves of rice plants were taken and measured on the upper, middle, and lower leaves with a SPAD 502 chlorophyll content analyzer.
(6) Root-to-shoot ratio = underground dry weight/aboveground dry weight.
(7) Phosphorus accumulation (mg/plant) = phosphorus content × dry weight.
(8) Phosphorus accumulation per plant (mg/plant) = aboveground phosphorus accumulation + underground phosphorus accumulation.
(9) Aboveground phosphorus utilization efficiency (g.g^−1^) = aboveground dry weight/aboveground phosphorus accumulation.
(10) Utilization efficiency of underground phosphorus (g.g^−1^) = underground dry weight/underground phosphorus accumulation.
(11) Phosphorus utilization efficiency per plant (g.g^−1^) = dry weight per plant/phosphorus accumulation per plant.

### 4.4. Data Processing and Analysis

Excel 2010 was used for preliminary data statistics and sorting, and SPSS 22.0 was used for correlation analysis, principal component analysis, and variance analysis. The single-factor analysis of variance (ANOVA) model was used in the experiment to analyze the effects of different phosphorus concentrations on the growth of rice. The Shapiro–Wilk test was employed to verify whether the plant height data conformed to the normal distribution (*p* > 0.05 indicates that the normal distribution is satisfied), and the Levene test was used to assess the homogeneity of variance (*p* > 0.05 indicates that the variances of each group are equal). The statistical analysis was carried out using SPSS 26.0 software, and the significance level was set as α = 0.05. If the ANOVA results showed significant differences among groups (*p* < 0.05), the Tukey HSD test was further adopted for comparison. The membership function method was used to comprehensively evaluate the low-phosphorus tolerance of 156 rice germplasms. Origin and the ggtree package in R were used to create correlation heatmaps and cluster analysis circular diagrams.

The relevant calculation formulas are as follows:

Low phosphorus tolerance coefficient = measured value of low phosphorus/measured value of norm(1)

(2) $$\mu \left(Xij\right)=\left(Xij-Xjmin\right)/\left(Xjmax-Xjmin\right)\;\;\;\;\;\;j=1,\;2,\;3,\;,\;n$$

where *µ* (*Xij*) represents the membership function value of the *j*th comprehensive index of the *i*th germplasm, *Xij* represents the *j*th comprehensive index of the *i-th* germplasm, *Xjmax* represents the maximum value of the *j*th comprehensive index, and *Xjmin* represents the *j*th minimum value of a comprehensive indicator.

(3) $$Wj=Pj/\sum_{j=1}^{m}P\;\;\;\;\;\;\;\;\;\;\;j=1,\;2,\;3,\ldots ,\;n$$

where *Wj* represents the importance and weight of the *j*th comprehensive index among all comprehensive indexes and *Pj* is the contribution rate of the *j*th comprehensive index of each variety.

(4) $$D=\sum_{j=1}^{m}\left[\mu (Xij)\times Wj\right]\;\;\;\;\;\;\;\;\;j=1,\;2,\;3,\ldots ,\;n$$

In the formula, D represents the comprehensive evaluation value of low phosphorus tolerance of various varieties under low phosphorus stress.

## 5. Conclusions

This study investigated the variations in morpho-physiological traits, root morphology, and material content of 156 different rice germplasm materials under normal and low phosphorus levels. The following conclusions are drawn. Under low-phosphorus conditions, the aboveground growth and development of rice germplasms were inhibited to varying degrees. Rice also exhibits different physiological responses to adapt to low-phosphorus conditions. To absorb more phosphorus for its own utilization, rice increases its root length, root surface area, and underground fresh weight. Through the analysis of the coefficient of variation, it is concluded that 16 indicators can be used as screening indicators [36]. Through the analysis of the coefficient of variation of each indicator and factor analysis, it is known that 16 indicators can be used as screening criteria for low phosphorus tolerance in rice seedlings. A principal component analysis was conducted on the low-phosphorus tolerance indices of various indicators, extracting a total of five principal components. A membership function analysis was used to determine the membership values and comprehensive score *D* values for each variety. Based on the cluster analysis diagram, the varieties could be classified into four categories. Scatter plots were generated using the comprehensive phosphorus efficiency values of different rice germplasms under normal-phosphorus and low-phosphorus conditions, the varieties could be classified into four categories. Through a comprehensive analysis of the cluster analysis diagram and scatter plot, the germplasm resources with strong low-phosphorus tolerance were ultimately identified as Y2-14, Y2-35, Y2-21, Jinongda 705, Changjing 625, and Jinongda 873, while those with poor low-phosphorus tolerance were identified as Jijing 338, Jingu 981, Tong 35, Y2-31, and Longdao 20. Currently, the hydroponic research conducted has achieved phased results, clearly demonstrating the low phosphorus tolerance performance of different rice germplasms under specific hydroponic conditions. These findings provide critical evidence and strong support for the in-depth exploration of rice’s low phosphorus tolerance and the determination of screening indicators. However, we must clearly point out that the field environment is highly complex and uncontrollable, which differs from the hydroponic environment. Subsequent in-depth re-evaluation of the agronomic performance of this rice germplasm under field conditions is required. After comprehensive consideration of various field factors, a rigorous and reliable statement on the true agronomic performance of the 156 different rice germplasm accessions can be made.

## Figures and Tables

**Table 1:** Variation ranges and variation coefficients of low-phosphorus tolerance coefficients for some rice traits.

Relative Traits of Rice	Variation Range	Average	Coefficient of Variation (%)
Relative plant height	0.481–0.919	0.611	13.59
Relative leaf area	0.702–1.023	0.914	8.50
Relative SPAD	0.536–0.87	0.726	6.83
Relative fresh weight above ground	0.397–0.892	0.649	39.45
Relative underground fresh weight	0.539–1.08	0.859	10.71
Relative total fresh weight per plant	0.497–1.24	0.906	21.29
Relative above ground dry weight	0.427–0.893	0.759	36.1
Relative underground dry weight	0.269–0.854	0.608	36.36
Relative total dry weight per plant	0.293–0.863	0.604	38.48
Relative aboveground phosphorus content	0.05–0.265	0.175	12.69
Relative underground phosphorus content	0.163–0.879	0.547	22.96
Relative aboveground phosphorus accumulation	0.32–0.565	0.424	14.79
Relative underground phosphorus accumulation	0.08–0.564	0.473	16.39
Relative phosphorus accumulation per plant	0.119–0.863	0.629	29.66
Relative aboveground phosphorus use efficiency	0.05–0.438	0.235	20.57
Relative underground phosphorus utilization efficiency	0.163–0.583	0.352	9.37
Relative phosphorus utilization efficiency per plant	0.147–0.845	0.563	39.97
Relative root–shoot ratio	0.299–1.252	0.899	6.08
Relative acid phosphatase	0.319–1.36	0.916	36.48
Relative root length	0.314–1.027	1.084	32.24
Relative root surface area	0.284–1.288	1.08	9.32
Relative root volume	0.162–1.26	1.163	5.02
Relative mean diameter	0.108–0.124	0.0703	9.03

**Table 2:** Characteristic values and contribution rates of some indicators.

Principal Component	Eigenvalue	Contributive Ratio (%)	Cumulative Contributive Ratio (%)
1	6.78	42.377	42.377
2	2.781	17.384	59.761
3	1.843	11.521	71.282
4	1.313	8.207	79.488
5	1.193	7.458	86.947

**Table 3:** Matrix of factor loading.

Low-P-Tolerant Index	1	2	3	4	5
Plant height	0.141	−0.024	0.002	0.049	−0.118
Fresh weight above ground	0.056	−0.013	0.337	−0.011	−0.379
Fresh underground weight	0.045	−0.112	0.306	−0.05	0.55
Total fresh weight per plant	0.069	−0.103	0.418	−0.032	0.245
Aboveground dry weight	0.138	−0.028	−0.012	0.068	−0.161
Underground dry weight	0.004	0.338	0.135	0.001	−0.013
Total dry weight per plant	0.122	0.144	0.08	0.066	−0.097
The amount of phosphorus in the ground	0.121	−0.015	−0.196	−0.031	0.145
Phosphorus content per plant	0.098	0.038	−0.224	−0.151	0.307
Ground phosphorus accumulation	0.139	−0.02	−0.099	0.019	−0.028
Underground phosphorus accumulation	0.032	0.302	−0.022	−0.11	0.202
Phosphorus accumulation per plant	0.142	0.083	−0.091	−0.039	0.096
Aboveground phosphorus utilization efficiency	0.021	0.048	−0.008	0.586	0.171
Utilization efficiency per plant	−0.012	0.012	−0.01	0.603	0.048
Root–shoot ratio	−0.054	0.311	0.144	−0.032	−0.005
Acid phosphatase	0.126	−0.025	0.087	−0.008	−0.29

**Table 4:** Comprehensive value of phosphorus efficiency of rice seedling under different phosphorus supply conditions (*D*).

Variety Number	NP	LP	Variety Number	NP	LP	Variety Number	NP	LP	Variety Number	NP	LP
V1	0.4702	0.4065	V40	0.3004	0.1378	V79	0.3459	0.3076	V118	0.3373	0.1531
V2	0.3503	0.3205	V41	0.4762	0.3771	V80	0.7149	0.5023	V119	0.2059	0.1222
V3	0.2304	0.1633	V42	0.4679	0.2906	V81	0.3335	0.3760	V120	0.1949	0.1213
V4	0.3623	0.4004	V43	0.3606	0.3040	V82	0.3089	0.2711	V121	0.4542	0.4766
V5	0.3088	0.2257	V44	0.5800	0.5105	V83	0.4451	0.4679	V122	0.6159	0.4481
V6	0.5343	0.5664	V45	0.5785	0.2948	V84	0.4686	0.3000	V123	0.6625	0.3183
V7	0.2343	0.3957	V46	0.5397	0.2906	V85	0.4714	0.3743	V124	0.3383	0.1554
V8	0.4507	0.5685	V47	0.4849	0.2896	V86	0.4665	0.3776	V125	0.5239	0.4561
V9	0.3493	0.2740	V48	0.5880	0.2927	V87	0.4601	0.2905	V126	0.5448	0.3014
V10	0.4732	0.4969	V49	0.3889	0.2839	V88	0.4738	0.2756	V127	0.5555	0.3879
V11	0.3568	0.3671	V50	0.5234	0.2955	V89	0.6300	0.3662	V128	0.4553	0.3609
V12	0.4114	0.2974	V51	0.2988	0.2720	V90	0.6652	0.2915	V129	0.3094	0.3034
V13	0.3264	0.3366	V52	0.3061	0.1693	V91	0.4380	0.3770	V130	0.2295	0.1266
V14	0.3575	0.3980	V53	0.4159	0.3387	V92	0.3020	0.1439	V131	0.7073	0.4475
V15	0.3633	0.2710	V54	0.5380	0.3061	V93	0.2084	0.1527	V132	0.4627	0.3136
V16	0.4560	0.4716	V55	0.4702	0.3746	V94	0.5240	0.4896	V133	0.4815	0.1400
V17	0.4549	0.4383	V56	0.2834	0.1080	V95	0.3255	0.2234	V134	0.4391	0.2886
V18	0.3548	0.2941	V57	0.4893	0.3625	V96	0.4830	0.2781	V135	0.3900	0.2745
V19	0.5363	0.4290	V58	0.3516	0.3734	V97	0.4002	0.3276	V136	0.2326	0.1488
V20	0.4473	0.3971	V59	0.2952	0.1568	V98	0.3611	0.4038	V137	0.4618	0.3033
V21	0.4811	0.3773	V60	0.4156	0.1140	V99	0.3048	0.1544	V138	0.2885	0.2263
V22	0.4758	0.2892	V61	0.5317	0.2264	V100	0.4470	0.2235	V139	0.3298	0.2817
V23	0.5196	0.4428	V62	0.3344	0.2883	V101	0.6929	0.4817	V140	0.1945	0.3009
V24	0.3340	0.2776	V63	0.3327	0.3138	V102	0.4560	0.2863	V141	0.2829	0.1430
V25	0.3233	0.3691	V64	0.3503	0.2992	V103	0.4552	0.2794	V142	0.2102	0.1307
V26	0.3631	0.3759	V65	0.2338	0.3195	V104	0.5584	0.3640	V143	0.2353	0.1697
V27	0.3430	0.2871	V66	0.4563	0.2258	V105	0.3938	0.3621	V144	0.3021	0.2958
V28	0.4109	0.2214	V67	0.4491	0.4643	V106	0.5258	0.4808	V145	0.3425	0.2215
V29	0.4548	0.3946	V68	0.3954	0.3625	V107	0.5811	0.4671	V146	0.3950	0.3203
V30	0.6714	0.4561	V69	0.4124	0.4432	V108	0.5298	0.4354	V147	0.3163	0.3197
V31	0.3100	0.2900	V70	0.5227	0.2878	V109	0.6123	0.5342	V148	0.4392	0.5041
V32	0.3232	0.2883	V71	0.4568	0.4653	V110	0.4626	0.4704	V149	0.4157	0.4469
V33	0.5882	0.4472	V72	0.3510	0.4475	V111	0.3062	0.2820	V150	0.4503	0.4968
V34	0.6015	0.4950	V73	0.3922	0.4805	V112	0.2790	0.2861	V151	0.5835	0.4959
V35	0.3671	0.2908	V74	0.2994	0.2257	V113	0.2974	0.2982	V152	0.4795	0.4400
V36	0.4002	0.2907	V75	0.4749	0.4804	V114	0.2360	0.1520	V153	0.3886	0.4501
V37	0.3884	0.2842	V76	0.4741	0.2708	V115	0.3051	0.2847	V154	0.4541	0.3173
V38	0.3232	0.1424	V77	0.3032	0.3071	V116	0.3133	0.3020	V155	0.5964	0.3868
V39	0.3409	0.3048	V78	0.6850	0.4478	V117	0.1875	0.1104	V156	0.4378	0.2898

**Table 5:** Kimura B nutrient solution formula.

Chemical	Mother Liquor (mmol/L)	Experimental Concentration (mmol/L)
(NH_4_)_2_SO_4_	364.8	0.3648
KH_2_PO_4_	182.2	0.1822
KNO_3_	183.0	0.1830
K_2_SO_4_	91.2	0.0912
Ca (NO_3_)_2_/Ca (NO_3_)_2_·4H_2_O	365.0/349.7	0.3650/0.3497
MgSO_4_/MgSO_4_·7H_2_O	547.5/547.0	0.5475/0.5470
H_3_BO_3_	30.1	0.0301
CuSO_4_·5H_2_O	0.3	0.0003
ZnSO_4_·7H_2_O	0.8	0.0008
MnCl_2_·4H_2_O	9.1	0.0091
(NH_4_) _2_MoO_4_·4H_2_O	0.5	0.0005
Na_2_·EDTA	22.2	0.0222
FeSO_4_·7H_2_O	20.0	0.0200

Note: The Kimura B nutrient solution needs to be diluted 1000-fold when in use.

## Data Availability

The original contributions presented in the study are included in this article; further inquiries can be directed to the corresponding authors.

## Supplementary Materials

The following supporting information can be downloaded at: https://www.mdpi.com/article/10.3390/plants14101543/s1. Table S1: Traits of 156 rice varieties grown low and normal P environment; Table S2: The comprehensive traits index values, weights, μ(X) and comprehensive evaluation value (D) of each variety; Table S3: Test rice Germplasm.
